# Anthocyanin metabolism in *Nelumbo*: translational and post-translational regulation control transcription

**DOI:** 10.1186/s12870-023-04068-3

**Published:** 2023-01-30

**Authors:** Xiaojing Liu, Fengfeng Du, Linhe Sun, Jinfeng Li, Shaozhou Chen, Naiwei Li, Yajun Chang, Jian cui, Wen chen, Dongrui Yao

**Affiliations:** 1 Jiangsu Key Laboratory for the Research and Utilization of Plant Resources/Jiangsu Engineering Research Center of Aquatic Plant Resources and Water Environment Remediation, Institute of Botany, Jiangsu Province and Chinese Academy of Sciences, Nanjing, 210014 China; 2 Collaborative Innovation Center for Efficient and Green Production of Agriculture in Mountainous Areas of Zhejiang Province, College of Horticulture Science, Zhejiang A&F University, Zhejiang 311300 Hangzhou, China

**Keywords:** Anthocyanin, Proanthocyanidins, *Nelumbo*, Metabolic flux, Lotus

## Abstract

**Background:**

Lotus (*Nelumbo* Adans.) is used as an herbal medicine and the flowers are a source of natural flavonoids. ‘Da Sajin’, which was firstly found in the plateau area, is a natural mutant in flower color with red streamers dyeing around white petals.

**Results:**

The LC–MS-MS results showed that eight anthocyanin compounds, including cyanidin 3-*O*-glucoside, cyanidin 3-*O*-galactoside, malvidin 3-*O*-galactoside, and malvidin 3-*O*-glucoside, were differentially enriched in red-pigmented tissues of the petals, whereas most of these metabolites were undetected in white tissues of the petals. Transcriptome profiling indicated that the relative high expression levels of structural genes, such as *NnPAL*, *NnF3H*, and *NnANS*, was inconsistent with the low anthocyanin concentration in white tissues. Members of the *NnMYB* and *NnbHLH* transcription factor families were presumed to play a role in the metabolic flux in the anthocyanin and proanthocyanidin biosynthetic pathway. The expression model of translational initiation factor, ribosomal proteins and SKP1–CUL1–F-box protein complex related genes suggested an important role for translational and post-translational network in anthocyanin biosynthesis. In addition, pathway analysis indicated that light reaction or photo destruction might be an important external cause for floral color determination in lotus.

**Conclusions:**

In this study, it is supposed that the natural lotus mutant ‘Da Sajin’ may have originated from a red-flowered ancestor. Partial loss of anthocyanin pigments in petals may result from metabolic disorder caused by light destruction. This disorder is mainly regulated at post translation and translation level, resulting in a non-inherited phenotype. These results contribute to an improved understanding of anthocyanin metabolism in lotus, and indicate that the translational and post-translational regulatory network determines the metabolic flux of anthocyanins and proanthocyanidins under specific environmental conditions.

**Supplementary Information:**

The online version contains supplementary material available at 10.1186/s12870-023-04068-3.

## Introduction

Flavonoids are an important class of polyphenolic compounds synthesized in plants. The flavonoid biosynthesis pathway responds to diverse developmental and environmental signals, such as ultraviolet (UV) radiation, pathogen attack, wounding, and low temperature [1, 2]. The metabolites synthesized by the flavonoid pathway, such as anthocyanins, proanthocyanidins (PAs, also termed condensed tannins), and flavandiols, are produced by closely related but distinct branches of this pathway [3]. Anthocyanins are the most conspicuous class of secondary plant metabolites owing to the variety of pigments that color many flowers, fruits, and seeds [1]. In addition to the role of attracting pollinators, anthocyanins are synthesized to deter herbivores and for protection against damage from UV irradiation. Proanthocyanidins, affect the taste and astringency of many fruits, and are involved in defense response against biotic and abiotic stresses [4]. The branches of the flavonoid biosynthetic pathway that produce anthocyanins and PAs have been studied extensively in multiple plant species, including *Arabidopsis*, grape, peach, and white clover [1, 5].

The anthocyanin biosynthetic pathway is initiated with the metabolism of phenylalanine. Subsequent reactions steps are catalyzed by key enzymes coded by structural genes, such as chalcone synthase (CHS) and dihydroflavonol reductase (DFR). A wide variety of flavonoids, anthocyanins, and PAs are generated from reactions catalyzed by different enzymes using leucoanthocyanidin and anthocyanidins as common substrates [6]. With regard to the anthocyanin-specific pathway, reactions are catalyzed by anthocyanidin synthase (ANS) to generate anthocyanidins, which are subsequently modified by flavonoid 3-*O*-glucosyltransferase (UFGT) to generate stable anthocyanins. As for PA-specific branches, reactions are catalyzed by leucoanthocyanidin reductase (LAR) and anthocyanidin reductase (ANR) to generate 2,3-*trans*-flavan-3-ols and 2,3-*cis*-flavan-3-ol, respectively. Proanthocyanidins are condensed products of these flavan-3-ol subunits [7].

The flavonoid pathway branch that produces anthocyanins and PAs is regulated by MYB–bHLH–WDR (MBW) complexes at the transcriptional level [8]. Among the transcription factors contributing to these complexes, R2R3-MYB transcription factors have been widely documented in peach, and a network of transcriptional activators and repressors are reported to balance PA and anthocyanin accumulation [5]. In apple, a telomere-binding protein MdTRB1 enhanced the activation activity of MdMYB9 to its downstream genes, and positively modulated anthocyanin and proanthocyanidin accumulation [9]. In strawberry (*Fragaria* × *ananassa*) fruit, transient down-regulation of *FcMYB1* results in strong reduction of *ANR* and *LAR* transcript abundance, leading to a discolored phenotype and indicating that *FcMYB1* plays a role in regulating the important branching-point of anthocyanin/ proanthocyanidins biosynthesis [10].

Although most studies suggest that anthocyanin biosynthesis regulatory genes function at the transcriptional level, regulation at the level of protein translation and post-translation has also been reported [11, 12]. In wheat and maize, repression of gene expression at the translational level has been proposed as the mechanism for *Myc1* regulation of anthocyanin metabolism [13]. The translational regulation involves the 5′ leader sequence of the mRNA affecting translational efficiency [14, 15]. Recently, F-box genes were identified as post-translational regulators that control phenylalanine ammonia lyase (PAL) ubiquitination and proteolysis. In Arabidopsis, Kelch domain-containing F-box proteins (KFBs), which are a structural component of the E3 protein–ubiquitin ligase complex, negatively regulate phenylpropanoid biosynthesis by mediating ubiquitination and subsequent degradation of PAL [16]. Down-regulation of KFBs genes might significantly increase the production of flavonols, anthocyanins, and PAs, and enhances plant tolerance to UV irradiation [17].

Lotus (*Nelumbo* Adans.) is an important medicinal plant used in various traditional medicines. The genus flourished in the middle Albian Stage (approximately 113–100.5 million years ago) during the Cretaceous Period [18]. During Quaternary glaciations, Asia and North America provided important refuges for the survival of lotus [19]. Only two remaining species of lotus are extant, namely *N. nucifera* Gaertn. and *N. lutea* Willd. *Nelumbo nucifera* is distributed and cultivated mainly in several Asian countries and northern Australia, while *N. lutea*, also known as American lotus, is only found in North America [20]. Usaually, the leaves, rhizomes, seeds, flowers, and buds of lotus were used as food and medicines in China, Japan, Thailand and India. Furthermore, the flowers and pedicels are a natural source of powerful flavonoids with versatile biological activities, such as anti-cancer, anti-obesity, anti-inflammatory and anti-neurodegenerative effects [21, 22].

**Fig. 1:**
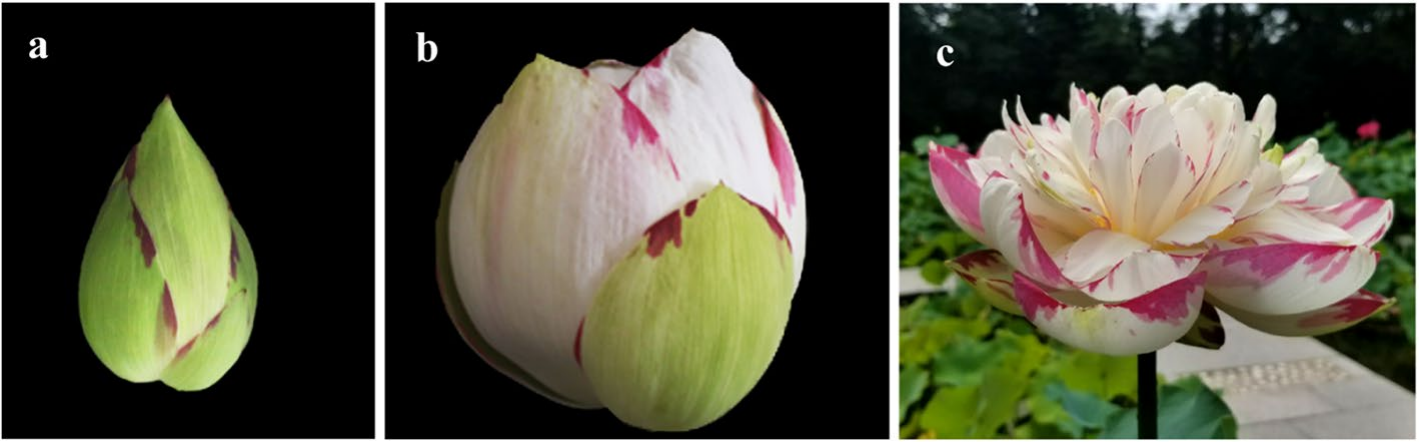
Flowers phenotype of Nelumbo ‘Da Sajin’. (a) flower bud; (b) flower at blooming day 1; (c) fully opened flower

‘Da Sajin’, a natural mutant of *N. nucifera*, was discovered in the Puzhehei area at an altitude of 1500 m in Yunnan province, China. The mutant is characterized by variegated flowers – the petals are predominantly white but have irregular areas with red pigmentation at the margins of the petals (Fig. 1). In this study, experiments were conducted to investigate the metabolic characteristics of the flavonoid pigments in the petals of ‘Da Sajin’. We proposed that: (I) The lotus ‘Da Sajin’ originated from a red-flowered ancestor, and the partial loss of anthocyanin might result from environmental changes. (II) Partial loss of anthocyanin in petals was not fully determined by the expression of structural genes or transcription factors, and translational or post-translational regulatory network influence the accumulation level of anthocyanidin. The results elucidate the mechanism of anthocyanin pigmentation in the petals of lotus and provide a foundation for the further regulation of flavonoid metabolism in lotus.

## Results

### Identification of anthocyanins in ‘Da Sajin’ petals

**Fig. 2:**
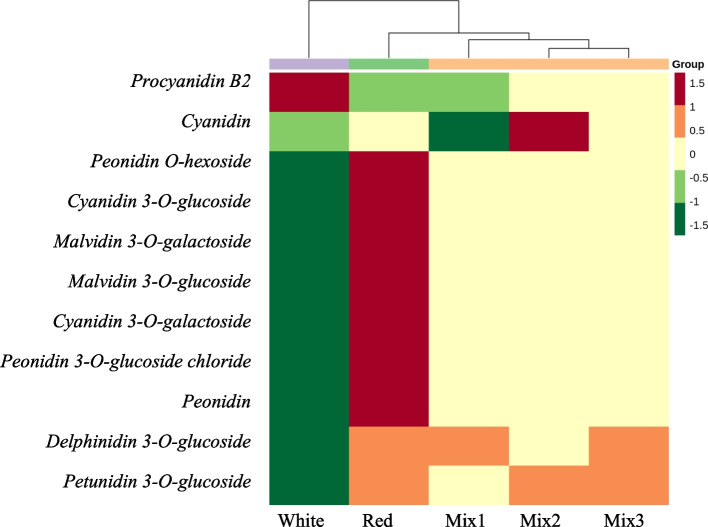
Hierarchical cluster analysis (HCA) of metabolite content in Nelumbo petals. The accumulation pattern of metabolites among different samples is clustered by R software after normalization. Mix 1, Mix 2 and Mix 3 were used as positive control. They are mix of anthocyanin standards

Red-pigmented patches are irregularly distributed on the petals of ‘Da Sajin’ (Fig. 1). Using LC–MS-MS, we determined the anthocyanin composition in the red (R) and white (W) portions of the petals. Anthocyanins with content fold changes of greater than 2 or less than 0.5 were defined as significantly changed metabolites. Nine significantly changed metabolites were detected between the red and white portions of the petals. The contents of cyanidin 3-*O*-glucoside, cyanidin 3-*O*-galactoside, malvidin 3-*O*-galactoside, malvidin 3-*O*-glucoside, peonidin 3-*O*-glucoside chloride, and peonidin *O*-hexoside were more than 10^6^ times higher in the red-pigmented areas, and these anthocyanins were almost undetected in the white portion of the petals (Table 1). Procyanidin B2 was also detected as a significantly changed metabolite but a relatively high content was detected in the white portion of the petals (Table 1). Hierarchical cluster analysis showed that the of content of these metabolites was significantly different in the white and red portions of the flower, indicating a physiological and genetic mechanisms underlying selective gene expression pattern in *Nelumbo* petals (Fig. 2).

**Table 1:** Anthocyanin composition in white and red portions of the petals of lotus 'Da Sajin' detected by LC–MS

	**Rt (min)**	**Molecular Weight (Da)**	**Ionization model**	**Compounds**	**Class**	**Fold change (R/W)**	**log**_**2**_** FC**
1	2.41	465.10	Protonated	Delphinidin 3-O-glucoside	Anthocyanins	3.20E + 02	8.32
2	2.55	449.10	Protonated	Cyanidin 3-O-glucoside	Anthocyanins	3.46E + 06	21.72
3	2.56	448.10	[M] +	Cyanidin 3-O-galactoside	Anthocyanins	4.52E + 06	22.11
4	2.61	479.00	Protonated	Petunidin 3-O-glucoside	Anthocyanins	9.82E + 02	9.94
5	2.77	493.00	Protonated	Malvidin 3-O-galactoside	Anthocyanins	1.78E + 06	20.77
6	2.79	498.09	[M-Cl] +	Peonidin 3-O-glucoside chloride	Anthocyanins	2.71E + 06	21.37
7	2.84	493.20	Protonated	Malvidin 3-O-glucoside	Anthocyanins	1.67E + 07	23.99
8	3.02	463.12	Protonated	Peonidin O-hexoside	Anthocyanins	3.02E + 06	21.53
9	3.04	578.14	[M + H] +	Procyanidin B2	Proanthocyanidins	4.64E-01	-1.11
10	3.37	287.24	Protonated	Cyanidin	Anthocyanins	1.14E + 00	0.19
11	3.94	301.10	Protonated	Peonidin	Anthocyanins	9.39E + 03	13.20

(*FC* fold change ≥ 2 or fold change ≤ 0.5)

### Transcriptome sequencing and metabolic pathway enrichment

To further understand the anthocyanin metabolic pathway in ‘Da Sajin’ flowers, the transcriptomes of the red and white portions of the flower bud petals were sequenced on an Illumina platform. Each sample comprised three biological replicates and thus six libraries were sequenced: red-pigmented tissues (samples R1, R2, and R3) and white tissues (W1, W2, and W3). Approximately 92.36%–96.73% of the total reads could be mapped to the reference genome (Table 2). For biological replicates of samples, edgeR was used for identification of differentially expressed genes. The screening criteria fold change ≥ 1.5 and adjusted *P*-value < 0.01 were applied to assign genes as differentially expressed. The fold change represents the ratio of gene expression values in two groups.

**Table 2:** Statistics for RNA-seq libraries generated from white and red portions of the petals of lotus 'Da Sajin' flowers mapped to the *Nelumbo nucifera* reference genome

BMK-ID	Total Reads	Clean-reads	Mapped Reads	Mapped-rate(%)
R1	41,183,692	41,183,692	38,498,515	93.48%
R2	40,801,074	40,801,074	37,683,871	92.36%
R3	51,127,462	51,127,462	48,704,020	95.26%
W1	42,967,556	42,967,556	41,562,516	96.73%
W2	46,894,078	46,894,078	45,065,208	96.10%
W3	43,228,390	43,228,390	41,732,687	96.54%

**Fig. 3:**
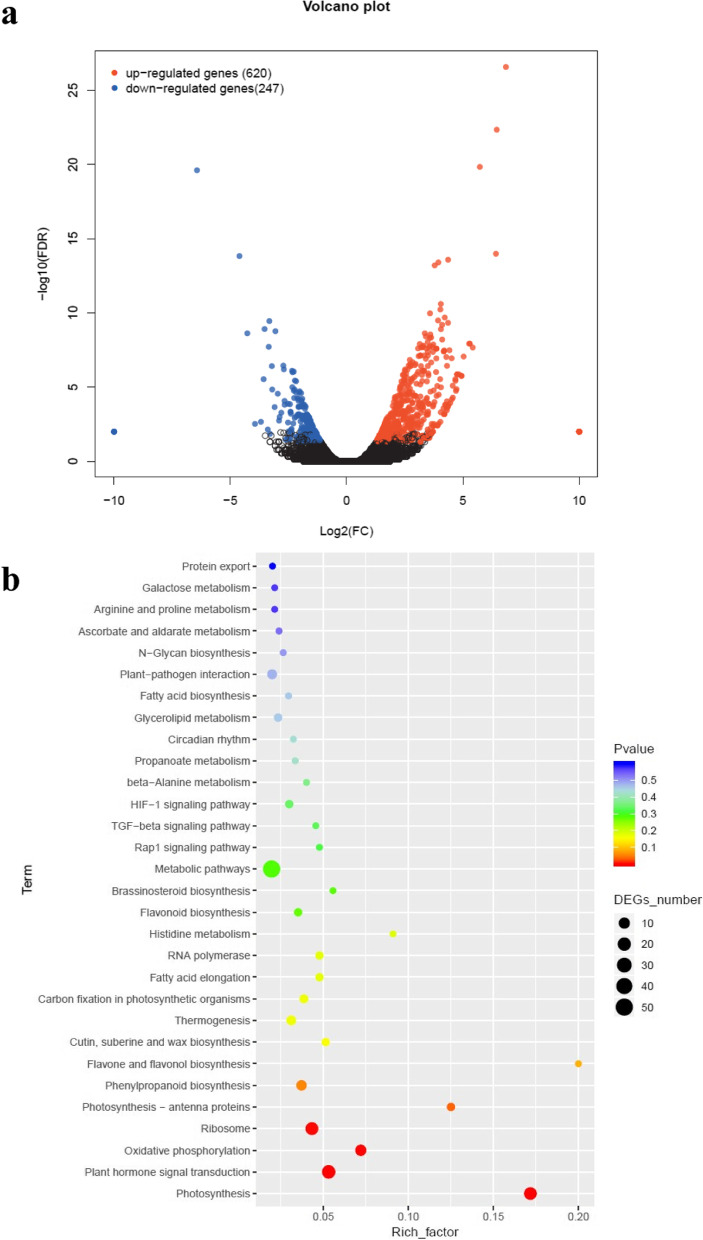
Volcano plot and enrichment of metabolic pathways identified by a KEGG analysis of differentially expressed genes between white and red portions of the petals of lotus ‘Da Sajin’. a. Volcano plot of differentially expressed genes. b. Enrichment of metabolic pathways by a KEGG analysis

A total of 867 significantly differentially expressed genes were detected between the red and white portions of the petals. Using red pigments as the control (W vs R), 620 genes were down-regulated, and 247 were up-regulated in the white portions of the petals (Fig. 3a). To explore the biological functions of these genes, we mapped the differentially expressed genes against terms in the KEGG database. Among the mapped pathways, the most highly enriched pathways were photosynthesis, plant hormone signal transduction, oxidative phosphorylation, ribosome, and photosynthesis– antenna proteins. In addition, phenylpropanoid biosynthesis, and flavone and flavonol biosynthesis were enriched; both pathways are directly associated with anthocyanin biosynthesis (Fig. 3b). Given that photosynthesis was identified as a significantly enriched pathway, we further checked the identity of the related genes. Photosynthesis-related genes, encoding photosystem I, photosystem II and antenna proteins, were up-regulated in the white portion of the petals (Table 3). Therefore, the red-pigmented tissues of the petals had stronger photosynthetic capacity than the white tissues owing to the difference in metabolites.

**Table 3:** Photosystem related genes differentially expressed between white and red pigments in ‘Da Sajin’ petals

Number	Gene symbol	log2FC (W vs R)	Type	Annotation
1	Nn2g14238	1.49	Up	photosystem I subunit O
2	Nn4g26289	1.20	Up	photosystem I reaction center subunit II, chloroplastic
3	Nn020s41489	-2.41	Down	photosystem II protein D1
4	Nn020s41494	-1.78	Down	photosystem II protein K
5	Nn020s41506	-1.89	Down	photosystem II protein D2
6	Nn049s41914	-1.90	Down	photosystem II phosphoprotein
7	Nn055s41927	-1.62	Down	photosystem I P700 apoprotein A2
8	Nn055s41932	-1.57	Down	photosystem I P700 apoprotein A1
9	Nn314s46301	-1.44	Down	photosystem II CP47 chlorophyll apoprotein

### Analysis of flavonoid biosynthesis genes

**Fig. 4:**
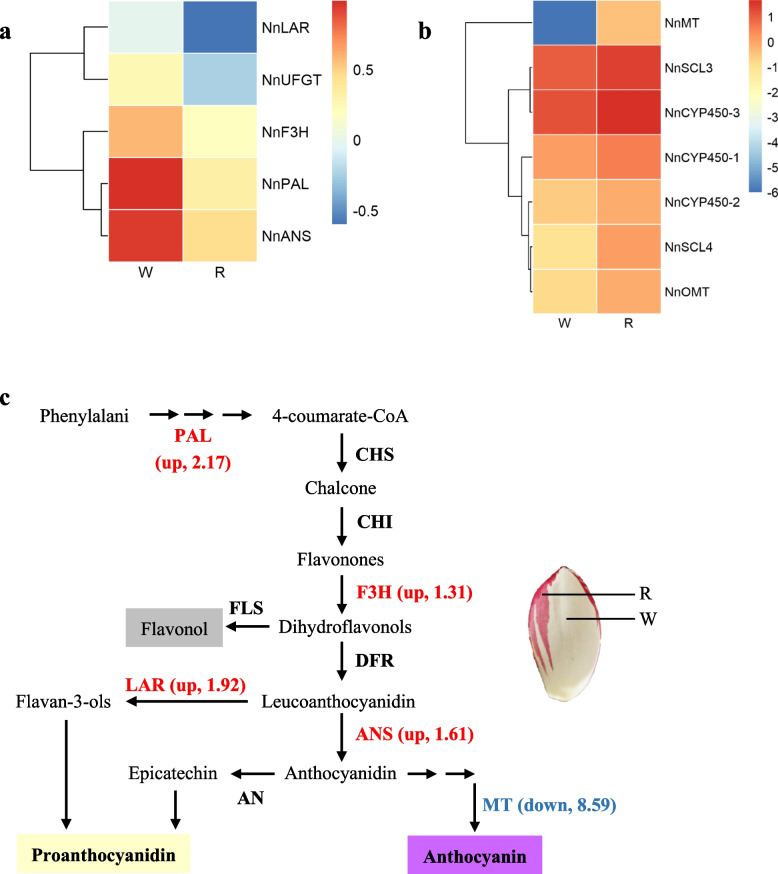
Relative expression of anthocyanin and proanthocyanidins related genes in white tissues of ‘Da Sajin’ petals. a. Heatmap of up-regulated structure genes in white tissues; b. Heatmap of down-regulated structure genes in white tissues; c. Graphic information of gene expression in anthocyanin biosynthetic pathway. The numbers represent the log2FC(W vs R). FC, Fold change; W, white tissues; R, red tissues. The gene in red represent up-regulated genes, and genes in blue represent down-regulated genes. The two closely-related pathway share common intermediates, and significantly down-regulation of NnMT and up-regulation of NnLAR may result in the diversion of metabolic flux away from anthocyanin production and toward colorless proanthocyanidin in white tissues of petals

To explore the mechanism of floral color pigmentation, specific attention was paid to the flavonoid biosynthesis pathway and the related genes. The differentially expressed flavonoid structural genes are shown in Fig. 4. Usually, increased expression of structural genes is expected and observed in organs with high anthocyanin concentration. Surprisingly, relatively high expression of anthocyanin structural genes, such as *NnPAL*, *NnF3H*, and *NnANS*, were detected in white portions of the petals (Fig. 4a). To understand the negative correlation between structural gene expression and anthocyanin pigmentation in the petals, genes involved in different branches of the flavonoid pathway were further analyzed to check the metabolic flux. Notably, expression of the flavonoid 3′,5′-methyltransferase gene, *NnMT*, was down-regulated by 8.59-fold in white tissues of ‘Da Sajin’ petals. In contrast, *NnLAR*, which is specifically involved in the proanthocyanin biosynthesis pathway, was significantly up regulated in the white portions of ‘Da Sajin’ petals (Table S1, Fig. 4a, c). Significant down-regulation of *NnMT* and up-regulation of the proanthocyanin-specific pathway genes may result in diversion of the metabolic flux away from anthocyanin production and toward colorless PA accumulation, which might be one reason for the absence of anthocyanin pigments in white portions of the petals.

### Analysis of transcription factors associated with the flavonoid pathway

**Fig. 5:**
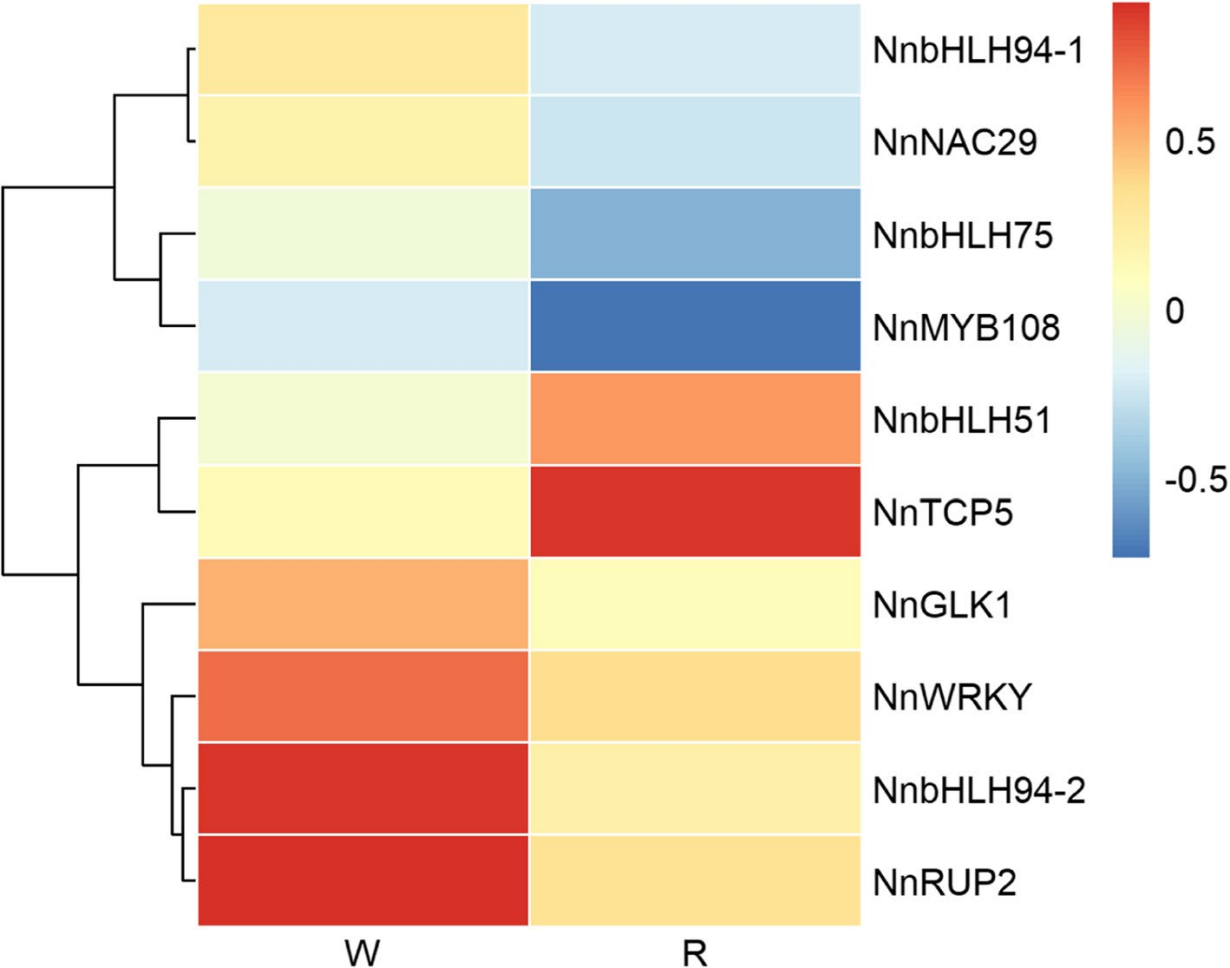
Heatmap of differentially expressed transcriptional factors in white and red tissues of ‘Da Sajin’ petals

It has been well documented that structural genes encoding enzymes of the anthocyanin and proanthocyanin pathways are regulated at the transcriptional level by MBW complexes [8]. Translational activators and repressors are both involved in the accumulation of anthocyanin and proanthocyanidin. In this study, a gene encoding a MYB108-like transcription factor was up-regulated in white portions of the petals. With regard to bHLH transcription factors, three genes were up-regulated and one was down-regulated in white tissues of the petals (Fig. 5, Table S2), indicating functional differentiation of their roles in the regulation of metabolic pathways.

### Analysis of translational regulators

**Fig. 6:**
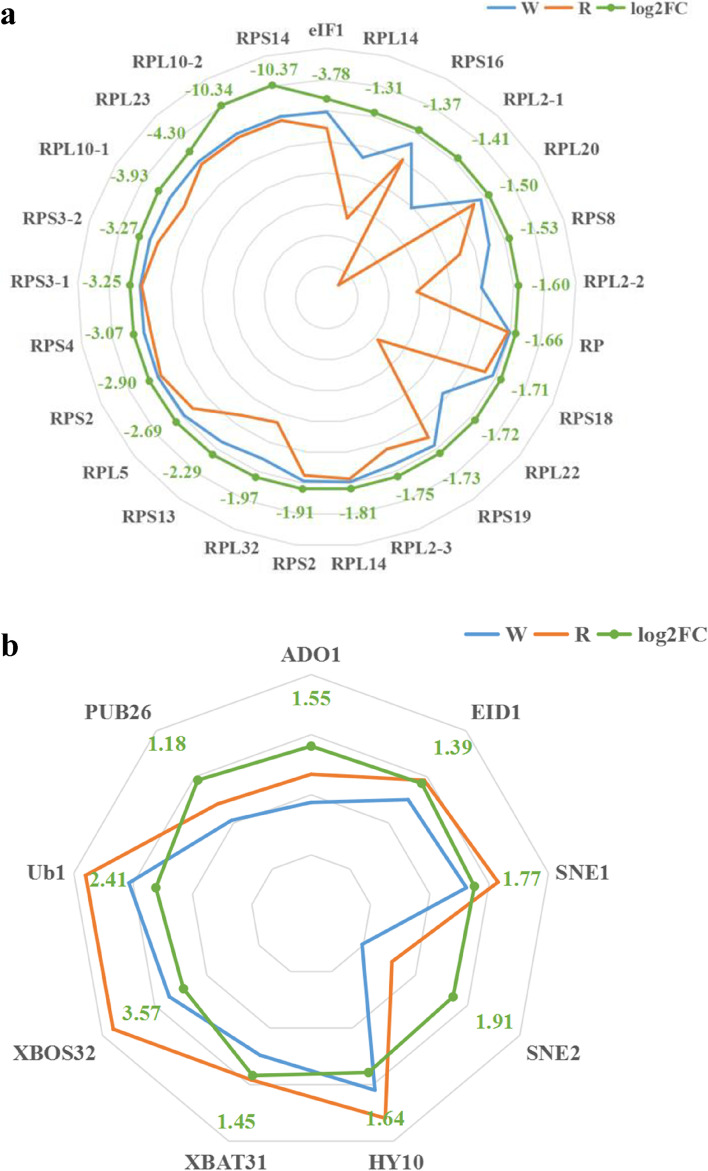
Map of translational and post-translational regulators differentially expressed between white and red portions of the petals of lotus ‘Da Sajin’. a. Translational initiation factor and ribosomal proteins. TPM (transcripts per million) is used to measure gene or transcript expression. W, white tissues; R, red tissues

Through screening the transcriptome data, the gene encoding translational initiation factor 1 (*NneIF1*) was significantly down-regulated in white tissues of the petals, with an expression level 3.78 times lower than that in red tissues. Twenty-four genes encoding ribosomal proteins, including the 30S, 50S, and 60S, were expressed at extremely low levels in white tissues of the petals (Fig. 6a and Table S3). Given that eukaryotic initiation factor 1 (eIF1) and ribosomal proteins are key mediators in selecting the correct initiation codon in the translation process, these results might suggest that specific transcripts are subject to precise translational regulation in the petals.

### Analysis of SCF complex-related genes

We noted that four F-box genes were differentially expressed between red and white tissues of the petals, and that the F-box genes showed relative high expression levels in white tissues. Among these F-box genes, Adagio protein 3 (a member of the ZTL protein family of blue-light photoreceptors) showed a relatively high expression level in the red tissues of the petals, which is 1.55 times than the level in the white tissues. Given that F-box proteins are structural components of the cullin-RING-based SKP1–CUL1–F-box protein (SCF) E3 ubiquitin–protein ligase complexes, genes associated with the SCF complex were further analyzed. Genes coding E3 ubiquitin–protein ligase, polyubiquitin, and U-box domain-containing protein were identified as up-regulated in the white portions of the petals (Fig. 6b and Table S4). These results indicated that F-box proteins might be post-translational regulators responsible for controlling flavonoid biosynthesis.

### Quantitative real-time PCR analysis

**Fig. 7:**
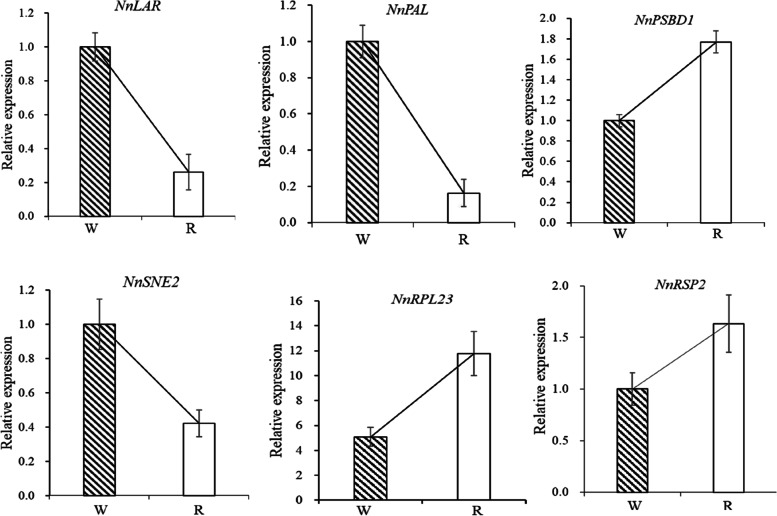
Analysis of gene expression model in Nelumbo ‘Da Sajin’ petals using quantitative RT-PCR. NnEF1α was used for normalization

Expression of several candidate genes were analyzed to validate the RNA-seq data using RT-qPCR. The structural genes *NnANR*, *NnLAR*, *NnDFR*, and *NnANS* were up-regulated, whereas the expression level of *NnUFGT* was comparatively low, in the white portions of the petals (Fig. 4). Generally, these findings were consistent with the RNA-seq results. As verified by RT-qPCR, a family of *NnMYB* transcription factors showed diverse expression patterns between red and white tissues in the petals, indicating that these MYBs might act as transcriptional activators or repressors, respectively, in the flavonoid biosynthesis pathway (Fig. 7).

## Discussion

Flower petals, which are reported to be chlorophylless, are comprised of non-photosynthetic tissues. Although the residual quantity of chloroplasts or chlorophyll were found in petal tissues, and the residual is presumed to have no physiological importance. However, photosynthetic activity has been confirmed in tobacco flowers, and the corolla photosynthetic apparatus shows the highest carbon metabolism at anthesis [23]. Furthermore, photoacoustic studies demonstrate that flower petals are photosynthetic and that chlorophylless photosynthesis is identified as an anoxygenic, anthocyanin-dependent process. Accumulation of anthocyanins results in additional light absorbance [24]. In the present study, photosynthesis was identified as the most enriched pathway and a majority of the photosynthesis-related genes were up-regulated in red tissues of the petals of ‘Da Sajin’ lotus (Table 3). This result provided experimental evidence for chlorophylless photosynthesis in the petals and it was indicated to be an anthocyanin-dependent process in lotus petals. Anthocyanin-dependent photosynthesis is negatively affected by radiation, and photodestruction of anthocyanin pigments occurs easily in high-radiation environments [25]. Therefore, we supposed that the natural lotus mutant ‘Da Sajin’, which is native to a plateau area, might have originated from a red-flowered ancestor, and the partial loss of anthocyanin pigments may result from environmental changes of photodestruction.

Anthocyanin pigments are generally determined by expression of structural genes [8]. Previous studies have shown that the pigments of lotus flowers may fall into the same categories. Proteomic analysis comparing red- and white-flowered lotus cultivars has indicated that expression of anthocyanin synthase (NnANS) and different methylation intensities of its promoter sequence determines anthocyanin accumulation in lotus flowers [26]. Sun et al. [27] observed that DFR, ANS, and UGT2 showed higher expression levels in yellow petals than in red petals, and the anthocyanin pigments were most likely to be determined by the variation of MYB5 transcription factors. In this study, eight types of anthocyanins were enriched in the red-pigmented tissues, whereas these metabolites were almost undetected in the white portions of ‘Da Sajin’ petals. However, anthocyanin accumulation was not determined by the structural genes in the anthocyanin-specific pathway (typically CHS, DFR, and ANS). The expression deficiency of *NnMT*, which is responsible for methylation of anthocyanidin, might result in accumulation of unmethylated anthocyanidin and a dramatic decrease in anthocyanin contents in the white tissues of the petals. In addition, up-regulation of proanthocyanin-specific pathway genes was detected in the white tissues of the petals. As anthocyanin and PAs share common intermediates, anthocyanins and PAs might be colocalized in epidermal cells of floral tissues in lotus, allowing the possibility of metabolic crosstalk within the flavonoid pathway. Thus, the two closely related pathways may be overlapped in epidermal cells of flower buds in lotus.

The present results raise questions about the organization or molecular regulation of the anthocyanin and PA pathways in petals of lotus. The anthocyanin and PA pathways are both regulated at the transcriptional level by MBW complexes [8]. With regard to MYB transcription factors, activators and repressors have both been reported in the regulation of anthocyanin and proanthocyanin accumulation [5]. According to the reports, high light induced anthocyanin pigment of *Petunia hybrida* is regulated by endogenous MYB transcription factors, not by bHLH or WD40 transcription factors [28]. MdMYB1 acts as a positive regulator of anthocyanin accumulation under high-intensity light in apple [29]. In lotus, MYB transcription factors might coordinately regulate the structural genes in the anthocyanin and PA pathways. However, whether these MYBs act as activators or repressors requires further experimental evidence. With respect to bHLH transcription factors, three genes were up-regulated in white tissues, and one gene showed relatively high expression in red portions of the petals, which was suggestive of functional diversification of the bHLH family in the regulation of the anthocyanin and PA branches. The present study offers the possibility of disrupting the MBW complex by regulation of MYB and bHLH transcription factors at the transcriptional level to balance anthocyanin and PA accumulation.

**Fig. 8:**
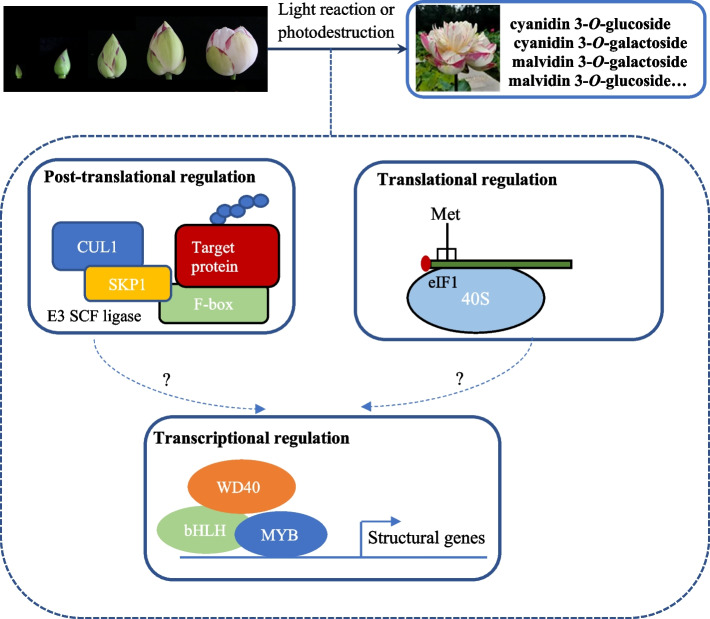
Proposed model of petal coloration in lotus ‘Da Sajin’. Dashes and question marks repents relations that still require further experimental verification

Post-transcriptional regulation of anthocyanin biosynthesis has been reported previously [30]. Translation initiation in eukaryotes begins with the small ribosome subunit loading with the help of eIF1, and then followed by successive ribosome scanning from the initiation codon. Eukaryotic initiation factor 1 (eIF1) is a crucial mediator of this process. Post-transcriptional regulation of anthocyanin biosynthesis regulators in wheat and maize involves the 5′ leader sequence of the mRNA affecting translational efficiency [14]. The wheat *TaMyc1* gene, which is a transcriptional factor containing a bHLH domain, governs anthocyanin metabolism in the pericarp of the grain. Regulation of *TaMyc1* at the mRNA translational level has been proposed to account for the synthesis of transcript variants differing in their 5′ leader sequences, resulting in differential translation efficiency among the transcripts [13]. In *Nelumbo*, significant down-regulation of translational initiation factor 1 (*NneIF1*) and up-regulation of 24 ribosome-related genes was detected in the white tissues of the petals, suggesting that they play a role in post-transcriptional regulation of the translation initiation process. The target of this precise translation regulation might be structural genes or transcription factors; however, experimental evidence is required to elucidate the potential mechanisms (Fig. 8).

Post-translational regulation mediated by F-box genes has also been reported to affect the accumulation of various phenylpropanoids, including anthocyanins, proanthocyanins and flavonols [16]. In *Chrysanthemum*, down-regulation of F-box genes is presumed to be a possible mechanism for anthocyanin accumulation in a mutant cultivar producing dark-purple ray florets [31]. Disruption of KFBs might significantly enhance the metabolic flux into the flavonoid biosynthetic pathway in Arabidopsis [16]. Recent studies have shown that a dramatic reduction in PAs content, to almost 40% of the wild-type PA content, is observed in KFB39-overexpression lines, whereas down-regulation of identified KFBs in Arabidopsis increased the accumulation of anthocyanins. Furthermore, expression of *KFB* genes is dramatically suppressed by exposure to UV-B radiation [17]. In the present study, a set of genes encoding F-box proteins, E3 ubiquitin–protein ligase and U-box domain-containing protein showed relatively high expression levels in the white portions of the petals. These results indicated that functional assembly of the SCF complex, and subsequent ubiquitylation and degradation of related proteins, occurred in lotus petals (Fig. 8). Furthermore, the targets for the SCF complex might be intermediate substrates of the flavonoid biosynthetic pathway or related transcription factors, and the subsequent ubiquitination and degradation of the target proteins might lead to reprogramming of the flavonoid biosynthesis pathway.

Therefore, the mechanism underlying anthocyanin metabolism in lotus was supposed to be a complex regulatory network involving both translational and post-translational regulators (Fig. 8). These regulators play an important role in transcriptional control of flavonoids biosynthetic genes and transcription factors, and thus influencing the metabolic pathway of flavonoids. In this study, coloration in lotus petals does not depend on the biosynthetic regulation of anthocyanidins, and the following modification and metabolic direction regulation may directly influence the flower color of lotus. Proanthocyanidins branches, which act as an important competitive pathway, affects the accumulation of anthocyanins in petals. In the biosynthesis process, anthocyanidins can be modified to anthocyanins or reduced to proanthocyanidins, which might be decided by translational and post-translational regulators. However, the precise regulation of the post-translational network involved in the regulation of flavonoid metabolism requires additional experimental evidence.

## Conclusions

In conclusion, the molecular mechanism underlying petal coloration in the natural lotus mutant ‘Da Sajin’ is proposed. Partial loss of anthocyanin pigments in petals may result from metabolic disorder, which involves a network of translational and post-translational regulators. The network mechanism might redirect the metabolic flux within the flavonoid biosynthesis pathway, diverting the metabolic flux away from anthocyanin biosynthesis and toward the accumulation of colorless PA in the petal.

## Methods

### Plant material

Lotus ‘Da Sajin’ plants were cultivated in a pond at the Institute of Botany, Jiangsu Province and Chinese Academy of Sciences. Flower buds were used as the experimental material (Fig. 1a). The petals were separated into red-pigmented and white portions. Preparations from these samples were used for LC–MS/MS analysis and transcriptome analysis.

### Extraction and LC–MS/MS analysis

Freeze-dried petals were used for samples extraction. The tissues were then crushed with a 400 MM mixer mill (Retsch, Germany). Extraction was carried out with about 100 mg powder using 1.0 mL 70% methanol aqueous solution overnight at 4 °C. After centrifugation at 10,000 g for 10 min, supernatants were filtered with a 0.22 μm pore size microporous membrane (SCAA-104, ANPEL). To screen the analytes, Shim-pack UFLC SHIMADZU CBM30A system were used for liquid Chromatography. The metabolites were separated by a column of ACQUITY UPLC HSS T3 C18. The eluent system was composed of water and acetonitrile solutions. The temperature was set to 40 °C, and the flow rate through the column was 0.40 mL/min. We use an API 6500 QTRAP LC/MS/MS with an ESI turbo ion spray interface system to characterize the metabolism.

### Transcriptome analysis

For total RNA extraction, the petals were extracted with Trizol Reagent (Invitrogen, Carlsbad, CA, USA). To construct the library, about 1 μg RNA per sample was used to generate sequencing libraries using NEBNext® Ultra™ RNA Library Prep Kit (NEB, Ipswich, MA, USA). Sequencing of the libraries was conducted with the Agilent 2100 Bioanalyzer system. The libraries were sequenced on Illumina platform. Paired-end reads were generated.

### Data analysis

Raw data were processed using in-house Perl scripts. After data processing, the clean reads were mapped to the *N. nucifera* reference genome (http://www.ncbi.nlm.nih.gov/genome/genomes/14095) using the HISAT2 software. We used edgeR for the differential expression analysis, and the *P*-values were adjusted by Benjamini–Hochberg approach. The genes, which were defined as differentially expressed genes (DGEs) have an adjusted *P*-value < 0.01. For KEGG analysis, the KOBAS software was used to determine the significantly enriched pathway [32].

### Quantitative real-time PCR analysis

To validate the RNA-sequencing (RNA-seq) data, RT-qPCR analysis was performed. Totally 1 µg RNA was used to synthesize cDNA. Then RT-qPCR analyses were performed using SYBR® *Premix Ex Taq*™ II (Takara Bio, Japan). Elongation factor 1-α (*EF1α*; accession number: XM_010255256) was used for normalization.

## Supplementary Information

**Additional file 1:** **Table S1.** Structural genes differentlyexpressed between white andred portions in petals. **Table S2.** Transcription factor genes differentially expressed between white and red portions of the petalsof lotus ‘Da Sajin’. **Table S3.**  Translational regulators differently expressedbetween white and red pigments in ‘DaSajin’ petals. **Table S4.**  Post-translational regulators differentlyexpressed between white and red pigments in ‘Da Sajin’ petals.

## Data Availability

The data that support the findings of this study have been deposited into CNGB Sequence Archive (CNSA) of China National GeneBank DataBase (CNGBdb) with accession number CNP0003743.
