# Supplementary material for: Anthocyanin metabolism in Nelumbo: translational and post-translational regulation control transcription
Source: BMC Plant Biol. 2023 Jan 30;23:61. doi: 10.1186/s12870-023-04068-3 (PMC9885672; doi:10.1186/s12870-023-04068-3)
Supplement: Supplementary file 1 — Additional file 1: Table S1. Structural genes differentlyexpressed between white andred portions in petals. Table S2. Transcription factor genes differentially expressed between white and red portions of the petalsof lotus ‘Da Sajin’. Table S3. Translational regulators differently expressedbetween white and red pigments in ‘DaSajin’ petals. Table S4. Post-translational regulators differentlyexpressed between white and red pigments in ‘Da Sajin’ petals. [file 12870_2023_4068_MOESM1_ESM.doc]

Table S1. Structural genes differently expressed between white and red portions in petals.

| **Number** | **Gene symbol** | **log2FC (W vs R)** | **Type** | **Annotation** | **Gene** |
| --- | --- | --- | --- | --- | --- |
| 1 | Nn4g24878 | 2.17 | Up | phenylalanine ammonia-lyase | *NnPAL* |
| 2 | Nn1g00388 | 1.31 | Up | flavonoid 3’-hydroxylase | *NnF3H* |
| 3 | Nn1g01275 | 1.61 | Up | anthocyanidin synthase | *NnANS* |
| 4 | Nn3g17713 | 1.92 | Up | leucoanthocyanidin reductase | *NnLAR* |
| 5 | Nn1g06876 | 1.61 | Up | UDP-glycosyltransferase | *NnUFGT* |
| 6 | Nn2g15849 | -8.59 | Down | flavonoid 3',5'-methyltransferase | *NnMT* |
| 7 | Nn3g17456 | 1.22 | Up | serine carboxypeptidase | *NnSCL1* |
| 8 | Nn8g39924 | 2.72 | Up | serine carboxypeptidase 24 | *NnSCL2* |
| 9 | Nn6g33376 | -1.34 | Down | serine carboxypeptidase II-3 | *NnSCL3* |
| 10 | Nn7g36340 | -3.63 | Down | serine carboxypeptidase 40 | *NnSCL4* |
| 11 | Nn1g08823 | -2.46 | Down | caffeic acid 3-O-methyltransferase | *NnOMT* |
| 12 | Nn8g39739 | -1.58 | Down | cytochrome P450 71A1 | *NnCYP450-1* |
| 13 | Nn3g20675 | -1.52 | Down | cytochrome P450 CYP72A219 | *NnCYP450-2* |
| 14 | Nn5g30405 | -1.64 | Down | cytochrome P450 86B1 | *NnCYP450-3* |

Table S2 Transcription factor genes differentially expressed between white and red portions of the petals of lotus ‘Da Sajin’.

| **Number** | **Gene symbol** | **log2FC**  **(W vs R)** | **Type** | **Annotation** |
| --- | --- | --- | --- | --- |
| 1 | Nn3g17881 | 1.72 | Up | transcription factor MYB108-like |
| 2 | Nn1g04233 | 1.27 | Up | transcription activator GLK1 |
| 3 | Nn1g08548 | 1.23 | Up | WRKY transcription factor 9 |
| 4 | Nn2g10913 | -1.98 | Down | transcription factor bHLH51-like |
| 5 | Nn3g17877 | 1.53 | Up | transcription factor bHLH75-like |
| 6 | Nn5g29978 | 1.63 | Up | transcription factor bHLH94-like |
| 7 | Nn6g33568 | 2.22 | Up | transcription factor bHLH94-like |
| 8 | Nn1g01438 | 1.93 | Up | WD repeat-containing protein RUP2 |
| 9 | Nn4g23242 | 1.48 | Up | NAC transcription factor 29 |
| 10 | Nn4g23449 | -2.52 | Down | transcription factor TCP5-like |

Table S3. Translational regulators differently expressed between white and red pigments in ‘Da Sajin’ petals.

| **Number** | **Gene symbol** | **log2FC**  **(W vs R)** | **Type** | **Annotation** | **Gene** |
| --- | --- | --- | --- | --- | --- |
| 1 | Nn021s41523 | -3.78 | Down | translational initiation factor 1 | eIF1 |
| 2 | Nn021s41521 | -1.31 | Down | ribosomal protein L14 | RPL14 |
| 3 | Nn020s41492 | -1.37 | Down | ribosomal protein S16 | RPS16 |
| 4 | Nn3g16151 | -1.41 | Down | 50S ribosomal protein L2 | RPL2-1 |
| 5 | Nn6g32081 | -1.50 | Down | ribosomal protein L20 | RPl20 |
| 6 | Nn021s41522 | -1.53 | Down | ribosomal protein S8 | RPS8 |
| 7 | Nn5g27212 | -1.60 | Down | 50S ribosomal protein L2 | RPL2-2 |
| 8 | Nn4g22336 | -1.66 | Down | 60S ribosomal protein | RP |
| 9 | Nn021s41531 | -1.71 | Down | ribosomal protein S18 | RPS18 |
| 10 | Nn021s41518 | -1.72 | Down | ribosomal protein L22 | RPL22 |
| 11 | Nn1g10001 | -1.73 | Down | ribosomal protein S19 | RPS19 |
| 12 | Nn020s41487 | -1.75 | Down | ribosomal protein L2 | RPL2-3 |
| 13 | Nn002s41362 | -1.81 | Down | 50S ribosomal protein L14 | RPL14 |
| 14 | Nn1g10086 | -1.91 | Down | ribosomal protein S2 | RPS2 |
| 15 | Nn064s42214 | -1.97 | Down | ribosomal protein L32 | RPL32 |
| 16 | Nn1g09985 | -2.29 | Down | ribosomal protein S13 | RPS13 |
| 17 | Nn1g09937 | -2.69 | Down | ribosomal protein L5 | RPL5 |
| 18 | Nn044s41810 | -2.90 | Down | ribosomal protein S2 | RPS2 |
| 19 | Nn055s41940 | -3.07 | Down | 30S ribosomal protein S4 | RPS4 |
| 20 | Nn4g25225 | -3.25 | Down | ribosomal protein S3 | RPS3-1 |
| 21 | Nn4g25226 | -3.27 | Down | ribosomal protein S3 | RPS3-2 |
| 22 | Nn1g09950 | -3.93 | Down | ribosomal protein L10 | RPL10-1 |
| 23 | Nn3g16152 | -4.30 | Down | 50S ribosomal protein L23 | RPL23 |
| 24 | Nn1g10162 | -10.34 | Down | ribosomal protein L10 | RPL10-2 |
| 25 | Nn287s46145 | -10.37 | Down | ribosomal protein S14 | RPS14 |

Table S4. Post-translational regulators differently expressed between white and red pigments in ‘Da Sajin’ petals.

| **Number** | **Gene symbol** | **log2FC**  **(W vs R)** | **Type** | **Annotation** |
| --- | --- | --- | --- | --- |
| 1 | Nn4g24402 | 1.55 | Up | adagio protein 3-like |
| 2 | Nn1g02149 | 1.39 | Up | EID1-like F-box protein 3 |
| 3 | Nn1g03222 | 1.77 | Up | F-box protein SNE-like |
| 4 | Nn1g07475 | 1.91 | Up | F-box protein SNE-like |
| 5 | Nn1g09124 | 1.64 | Up | protein LIGHT-DEPENDENT SHORT HYPOCOTYLS 10 |
| 6 | Nn5g26929 | 1.45 | Up | putative E3 ubiquitin-protein ligase XBAT31 |
| 7 | Nn5g31146 | 3.57 | Up | probable E3 ubiquitin-protein ligase XBOS32 |
| 8 | Nn8g41204 | 2.41 | Up | polyubiquitin-like |
| 9 | Nn5g30343 | 1.18 | Up | U-box domain-containing protein 26-like |
